# A Framework for AI-Assisted Detection of Patent Ductus Arteriosus from Neonatal Phonocardiogram

**DOI:** 10.3390/healthcare9020169

**Published:** 2021-02-05

**Authors:** Sergi Gómez-Quintana, Christoph E. Schwarz, Ihor Shelevytsky, Victoriya Shelevytska, Oksana Semenova, Andreea Factor, Emanuel Popovici, Andriy Temko

**Affiliations:** 1 Electrical and Electronic Engineering, University College Cork, T12 K8AF Cork, Ireland; 114220508@umail.ucc.ie (O.S.); e.popovici@ucc.ie (E.P.); atemko@ucc.ie (A.T.); 2 Irish Centre for Maternal and Child Health Research, University College Cork, T12 K8AF Cork, Ireland; christoph.schwarz@ucc.ie; 3 Faculty of Information Technologies, Kryvyi Rih Institute of Economics, 50479 Kryvyi Rih, Ukraine; sheleviv@gmail.com; 4 Faculty of Postgraduate Education, Dnipropetrovsk Medical Academy of Health, 49098 Dnipro, Ukraine; shelevika@gmail.com; 5 Department of Anatomy and Neuroscience, University College Cork, T12 K8AF Cork, Ireland; andreea.factor@ucc.ie

**Keywords:** patent ductus arteriosus, phonocardiogram, heart sound, neonates, congenital heart defects, machine learning, boosted decision trees

## Abstract

The current diagnosis of Congenital Heart Disease (CHD) in neonates relies on echocardiography. Its limited availability requires alternative screening procedures to prioritise newborns awaiting ultrasound. The routine screening for CHD is performed using a multidimensional clinical examination including (but not limited to) auscultation and pulse oximetry. While auscultation might be subjective with some heart abnormalities not always audible it increases the ability to detect heart defects. This work aims at developing an objective clinical decision support tool based on machine learning (ML) to facilitate differentiation of sounds with signatures of Patent Ductus Arteriosus (PDA)/CHDs, in clinical settings. The heart sounds are pre-processed and segmented, followed by feature extraction. The features are fed into a boosted decision tree classifier to estimate the probability of PDA or CHDs. Several mechanisms to combine information from different auscultation points, as well as consecutive sound cycles, are presented. The system is evaluated on a large clinical dataset of heart sounds from 265 term and late-preterm newborns recorded within the first six days of life. The developed system reaches an area under the curve (AUC) of 78% at detecting CHD and 77% at detecting PDA. The obtained results for PDA detection compare favourably with the level of accuracy achieved by an experienced neonatologist when assessed on the same cohort.

## 1. Introduction

Congenital Heart Defects (CHD) is malformations that occur due to abnormal development of the heart. These malformations can lead to a broad spectrum of clinical presentation, which implies a low or deficient performance of such a vital organ. Those diseases affect approximately 1% of newborns and account for 3% of all death among infants. Therefore, CHD is one of the most frequent causes of infant mortality [1]. In advanced well-resourced settings, most CHDs are detected by antenatal ultrasound, which allows detecting the heart pathology as early as 12–16 week of gestation. However, a significant portion of the heart anomalies stays undetected antenatally, and the diagnostic accuracy of antenatal diagnosis remains limited [2]. According to the retrospective data [3], the perinatal diagnostic rate of the CHDs is 39% during a 10 years period, with no increase during this period. The perinatal diagnostic rate of the critical CHDs (defined as potentially causing early death and requiring therapy in the neonatal period) is 50% (but can be as low as 13% and as high as 87%) [4]. Thanks to the introduction of special screening protocols, the sensitivity of the procedure for detecting complex CHDs increased from 29.8% to 88.3% [5,6]. The availability of antenatal and postnatal ultrasound is limited, and prioritisation is performed based on alternative more readily available methods. In resource-constraint settings, the availability of ultrasound is either scarce or nonexistent, and those alternative methods can potentially become even more advantageous in this context.

Auscultation is a part of the clinical routine examination in newborns. However, heart sound evaluation directly after birth can be challenging due to physiological shunt sounds through the Ductus Arteriosus. In the heart of the foetus, the role of Ductus Arteriosus is to allow the blood ejected by the right ventricle to bypass the lungs—a so-called right-to-left shunt. During the postnatal transition, the lung function changes starting with the first breath resulting in a shunt direction change during the normal transition to left-to-right. In the first 48 to 72 postnatal hours, Ductus Arteriosus closes in the vast majority of term infants [7,8,9]. However, delayed ductal closure beyond 72 h is considered pathological and becomes part of the CHD spectrum (patent ductus arteriosus, PDA) [10,11,12,13]. 

An early objective screening method based on heart sound assessment should differentiate between sounds with and without signatures of PDA and CHDs. This is of great importance, especially within the first three postnatal days, in particular, to reduce unnecessary examinations and optimally use limited echocardiography capacities without missing PDA dependent CHDs. While the auscultation is a low cost and reliable tool to screen for neonatal heart defects, heart sounds interpretation is subjective, dependent on the assessor’s hearing acuity and the acquired level of expertise. Assistance from artificial intelligence (AI) can fill the gap and provide an objective interpretation of heart sound, to complement the traditional auscultation method [14].

Thanks to the growing availability of data and computational power in recent years, AI and Machine Learning (ML) in particular, are becoming increasingly popular to solve many clinical healthcare problems, at times outperforming human decision-making [15,16]. ML can learn from large datasets and derive objective decisions, which are not influenced by individual perception, fatigue, or mood. 

A digital stethoscope can record heart sounds to lead phonocardiogram (PCG) recordings. Most works on automated PCG classification have been performed in the adult population where the data are easier to be acquired, with a few publicly available datasets [17]. Several automatic segmentation algorithms, features, classifiers, and metrics have been reviewed [18]. The 2016 PhysioNet challenge asked participants to classify PCG recordings between normal/abnormal conditions automatically. The authors of the winner solution of the challenge proposed to join the advantages of a neural network model that analysed raw data and a classical boosting classifier fed with time and frequency domain features [19]. The runner-up approach authors utilised a large set of different acoustic features extracted from various feature domains fed into a support vector machine (SVM) classifier [20]. 

Considerably fewer works address automated PCG classification in the paediatric population. An ML can accurately diagnose PCGs compared in the 3-class classification problem (no murmurs, innocent murmurs, and pathologic murmurs) on a cohort of 106 children with an average age of 8 years old has been demonstrated in [21]. The frequency band analysis of paediatric PCG with SVM for murmur characterisation was performed in [22], with the focus on the Android app development towards clinical usage of the method. 

Even fewer works have addressed the problem of automated interpretation of PCG in newborns. A statistical analysis of various features was performed towards automated detection of PDA murmur in a small cohort of 25 preterm infants [23]. Differentiation between healthy and pathological heart murmurs in newborns with a set of basic audio features, and an SVM classifier was performed with the main emphasis on automated PCG segmentation, denoising, and cycle selection [24].

Taking into account that ultrasound screening has limited availability in both antenatal and postnatal screening procedures and that clinical examination by auscultation lies on subjective interpretation, this study aims to provide an objective decision support by discriminating the sound clips with and without signatures of PDA and CHD by means of ML. The proposed solution aims to improve early diagnosis of PDA or CHD, which will improve the efficiency of prioritisation of candidates for ultrasound assessment and improve the outcomes for treatment. 

The specific contributions of this study are:

- The ML-based solution is designed to address two clinical problems, CHD vs. healthy, and PDA vs. healthy.
- The solutions are validated on a comprehensive real clinical dataset composed of heart sound recordings from a total of 265 newborns.
- The importance of various features and auscultation points is assessed for the task.
- The designed ML-based method to identify the presence of PDA signature in a sound clip is contrasted against the ability of an experienced neonatologist to do the same.

## 2. Materials and Methods

**Figure 1:**
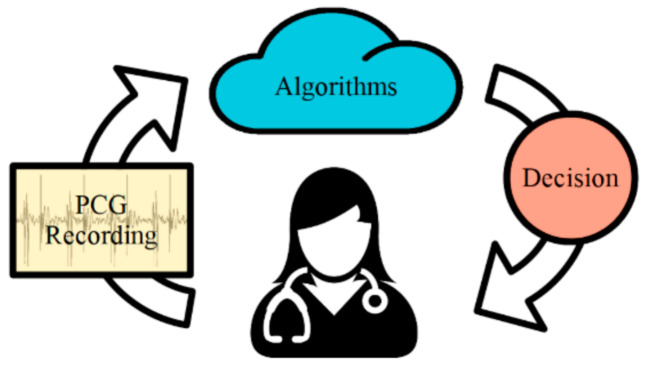
Cloud-based service scheme using phonocardiogram (PCG).

The deployment of the heart abnormality detection system developed in this study as a cloud-based objective decision support system is shown in Figure 1. The heart sounds are first uploaded to a cloud where the classification algorithm processes them with the decisions and confidence fed back to the user (physician).

**Figure 2:**
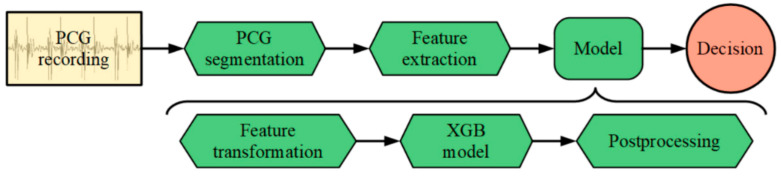
The pipeline of phonocardiogram (PCG) processing by artificial intelligence (using XGBoost).

The block diagram, with various stages of the classification algorithm, is shown in Figure 2. The algorithm can be divided into three main parts. First, each PCG recording goes through the segmentation process, where the PCG signal is segmented into separate heartbeat cycles, with each cycle further segmented into the four consecutive parts—S1, systole, S2, diastole. After that, a set of 200 features is extracted from each cycle. This feature set is then fed into an ensemble of boosted decision tree models, with the model outputs post-processed to lead the final decision. The detailed description of the developed system is described below.

### 2.1. Dataset

The dataset used in this study was collected between September 2013 and September 2018 at two hospitals in Ukraine: Kharkiv City Perinatal Center (Centre 1) and Maternity Hospital Nº1 of Kryvyi Rih (Centre 2). Informed parental consent was obtained for every participant before study inclusion. The study was approved by local ethics committees (Dnipropetrovsk medical academy Bioethics Committee, approval #1 dated 11/01/2013; Kryviy Rih City Clinical Maternity Hospital Ethics Committee, approvals #2, #3 dated 10/01/2014 and 10/01/2017, Kharkiv City Perinatal Center Bioethics Committee, approval #1 dated 18/06/2018).

In total, 265 newborns were included in the study, with the gestational ages ranging between 35 and 42 weeks. All patients had their diagnosis (healthy, PDA, or CHD) confirmed by echocardiography. Table 1 presents detailed demographic and neonatal variables of the dataset. 

All patients were clinically healthy newborns at the time of the auscultation recording with no vivid signs of the CHD or pulmonary hypertension. In most cases, the diastolic murmurs developed later as the disease progressed and pulmonary hypertension developed, at that point the CHD can be suspected with other routine methods. The samples used in this study have no clinically detected diastolic murmurs, which would be of clinical significance for the early screening. However, 15 patients had systolic murmurs classified as physiological or innocent, according to echocardiography results. Consequently, those patients were categorised into the healthy group. Out of 265 patients in the database, there were nine patients with both PDA and CHD confirmed by the ultrasound, but for simplicity, this study categorises each patient within a single diagnosis group, either CHD, PDA, or healthy. Patients with both CHD and PDA were assigned into the CHD group due to a higher priority of such diagnosis. 

**Figure 3:**
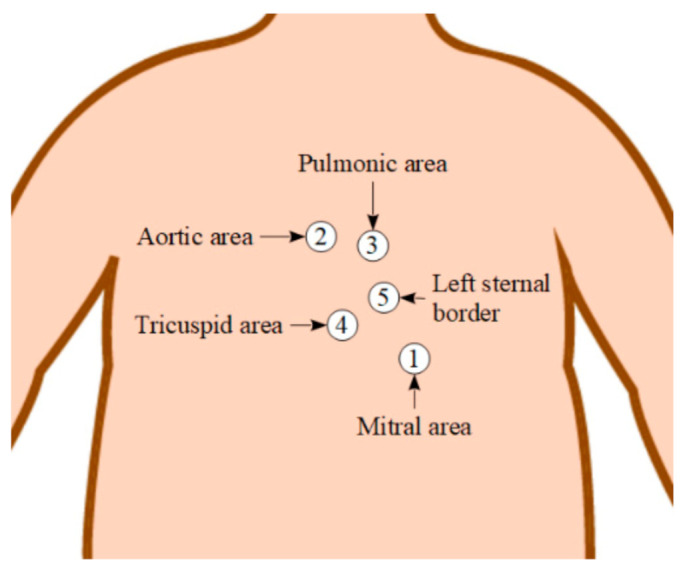
Approximate location, names, and chosen numeration of cardiac auscultation areas on the torso.

For each patient, PCG recordings were taken within the first six days of life from the five auscultation points shown in Figure 3, using a digital stethoscope recording audio at 44.1 kHz and 16 bit resolution (Thinklabs ds32a and ThinkLabs One, Centennial, CO, USA). The dataset used in this study consists of 265 PCG recordings of a total length of 7 h 48 min.

### 2.2. PCG Signal Segmentation

**Figure 4:**
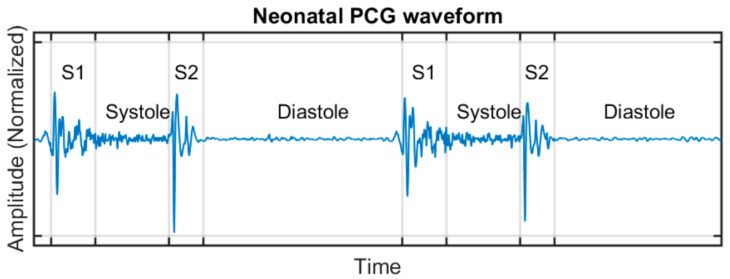
Two consecutive cardiac cycles showing a phonocardiogram waveform along with its four sound phases: S1, systole, S2, and diastole.

The normal heart sound consists of a cyclic sequence of two beats known as S1 and S2, producing the familiar “lub-dub” rhythmic sound that can be heard using a stethoscope applied to the patient chest. This sequence is driven by the cardiac cycle, which consists of alternating periods of heart contraction (systole) and relaxation (diastole). S1 is produced principally by vibrations created by the closure of the atrioventricular valves (mitral and tricuspid) located between the upper (atria) and lower (ventricles) chambers of the heart, at the beginning of ventricular systole. S2 is produced by vibrations created by the closure of the semilunar valves (aortic and pulmonary) in the arteries leading out of the ventricles at the end of the systole and beginning of the ventricular diastole. There is a relation between the PCG signal and the electrocardiogram signal (ECG). R-peak of ECG waveform matches the beginning of S1 sound in PCG and T-wave of ECG matches with the end of S2 sound [25]. An example of neonatal PCG is shown in Figure 4.

Other audible sounds that can be found during auscultation are murmurs. Those sounds are produced as a result of the turbulent flow of blood strong enough to produce audible noise. Heart murmurs are often signatures of heart valves’ pathological changes, and they are usually found during auscultation in primary healthcare. However, some murmurs are functional (innocent) in neonates and children, not any CHD. Murmurs can occur during systole or diastole intervals or continuously throughout the entire cardiac cycle.

Since five auscultation locations are used in this study (referred to as auscultation points or simply points, hereafter, in this manuscript) each location emphasises certain heart sound characteristics. The mitral area is the best place to listen to S1 and also the murmur of the mitral insufficiency (regurgitation), or mitral stenosis. The aortic area is suitable to listen to S2, as well as the murmur of aortic stenosis. The pulmonic area is suitable to detect the continuous murmur of PDA, as well as murmurs related to pulmonary stenosis and pulmonary insufficiency (regurgitation). The tricuspid area and the left sternal border are used to detect the murmurs of tricuspid stenosis and insufficiency (regurgitation), as well as the murmur of the ventricular septal defect [26].

In this study, the segmentation process has been performed manually: once the PCG recordings were made, those were manually segmented to heartbeat cycles and likewise each cycle into the four parts shown in Figure 4 (S1, systole, S2, and diastole). A minimum of two heartbeat cycles (but typically five) were selected from each auscultation point, resulting in a total of 10 to 27 single cycles per patient (22 on average). From the original dataset of 7 h 48 min long, after segmentation, the dataset from which the features were extracted consists of 5904 cycles from 265 patients, totalling 47 min 53 s of audio data fed into the feature extraction algorithm.

### 2.3. Feature Extraction

Different types of features are extracted from each of the four heart-sound intervals due to the difference in the amplitudes and structure of the PCG waveform in each interval, as reported in [20]. A total of 200 features were extracted (detailed in Table 2, Table 3 and Table 4) to capture the signal’s temporal, frequential and energy aspects. Some features are extracted from all four intervals (Table 2); others are extracted only from S1 and S2 (Table 3) or only from systole and diastole intervals (Table 4). Additionally, the average beats per minute (per auscultation point) and the relative cycle length were used as features. A few of the extracted features have been previously reported to be discriminative for neonatal PCG characterisation in [27], whereas others such as audio sub-band-specific energy and root mean squared (RMS) are introduced in this study for the first time. A large variety of features are intentionally designed to investigate which features are most important for the task. Before extracting all the mentioned features, the recordings were downsampled to 2 kHz since the maximum frequency considered on the whole feature set was 1 kHz.

### 2.4. Classification Algorithm: XGBoost

Boosting is a method of building an accurate classifier from the ensemble of “weak” learning algorithms [28]. Gradient boosting allows solving both regression and classification problems using a set of decision trees [29]. XGBoost is an open-source implementation of the regularised boosted decision trees [30]. This library has been successfully utilised in the winning solutions for several machine-learning competitions (Kaggle) and showed the state-of-the-art results on a vast array of problems. 

At each stage of gradient boosting, 1≤k≤K, a weak classifier, $f_{k}$, is generated. On the next stage, an improved classifier is constructed $f_{k+1}\left(x\right)\;=f_{k}\left(x\right)\;+\;h\left(x\right)$ by fitting *h* to the residuals, $y-f_{k}\left(x\right)$. To learn a set of functions (decision trees) on each iteration, the following objective function is optimised:

(1) $$Obj=\overset{N}{\underset{i=1}{\sum }}l\left({\hat{y}}_{i},y_{i}\right)+\overset{K}{\underset{k=1}{\sum }}\Omega \left(f_{k}\right)$$

Here, *N* is the number of training examples, *K* is the number of iterations, l is a differentiable convex loss function that measures the difference between the prediction ${\hat{y}}_{i}$ and the target $y_{i}$; Ω measures the complexity of the tree function, which allows avoiding overfitting by penalising complicated building models. The complexity of the tree is defined as follows:

(2) $$\Omega \left(f\right)=\gamma T+\frac{1}{2}\lambda \overset{T}{\underset{j=1}{\sum }}w_{j}^{2}$$

Each function, f, corresponds to an independent tree structure with a vector of leaf weights, w, on the jth leaf; T is the number of leaves in the tree. The number of terminal nodes is penalised with the γ parameter; weights optimisation is performed using L2 norm, to encourage leaves with smaller weighs.

The objective function is optimised using the second-order Taylor expansion and is defined as follows:

(3) $$Obj^{\left(t\right)}\approx \overset{N}{\underset{i=1}{\sum }}(g_{i}f_{t}\left(x_{i}\right)+\frac{1}{2}h_{i}f_{t}^{2}\left(x_{i}\right))+\Omega \left(f_{t}\right)$$

where $g_{i}=\partial_{{\hat{y}}_{i}^{\left(t-1\right)}}l\left({\hat{y}}_{i}^{\left(t-1\right)},y_{i}\right)$ and $h_{i}=\partial_{{\hat{y}}_{i}^{\left(t-1\right)}}^{2}l\left({\hat{y}}_{i}^{\left(t-1\right)},y_{i}\right)$ are derivatives and Hessian of the loss function at iteration, *t*; and $x_{i}$ is a data instance or datapoint (feature vector). The optimal weight, $w_{j}^{*}$, for the leaf, *j*, is obtained as follows:

(4) $$w_{j}^{*}=-\frac{\sum_{i\in I_{j}}g_{i}}{\sum_{i\in I_{j}}h_{i}+\lambda }$$

Each decision tree, $f_{t}$, is generated by making a decision on how to select and split features. This decision is performed using the gain parameter, which measures an improvement brought by each split.

(5) $$Gain=\frac{1}{2}\left[\frac{{\left(\sum_{i\in I_{L}}g_{i}\right)}^{2}}{\sum_{i\in I_{L}}h_{i}+\lambda }+\frac{{\left(\sum_{i\in I_{R}}g_{i}\right)}^{2}}{\sum_{i\in I_{R}}h_{i}+\lambda }-\frac{{\left(\sum_{i\in I}g_{i}\right)}^{2}}{\sum_{i\in I}h_{i}+\lambda }\right]-\gamma$$

Here, $I_{R}$ and $I_{L}$ are sets of instances in the left and right nodes after the split, γ is a regularisation parameter. It can be seen that is the resultant gain is smaller than parameter γ, the split is not added. In this study, the gain parameter was used to quantify the importance of each feature for the constructed tree ensemble.

XGBoost implements the following regularisation techniques: rows (training examples) and columns (features) subsampling, which introduce randomness to the learning process; and shrinkage (learning rate), which scales new weights by a factor *η* and leaves space for other trees to improve the model. 

The models were constructed using the following settings: objective = binary: logistic, eval_metric = auc, eta = 0.03 (learning rate). 

Other hyperparameters that control the complexity and regularisation of the model need to be tuned. These parameters are:

- *max_depth*: the number of branch levels for each decision tree;
- *subsample*: a ratio of randomly selected data rows or samples;
- *colsample_bytree*: a ratio of randomly selected data columns or features;
- *tree_num*: the number of decision trees used by the model.

From the trained model, a list of the most important features can be constructed. Since the large feature set is utilised in this work, one of the targets of the study was to select a smaller number of relevant features and maintain the same performance.

### 2.5. Model Evaluation

**Figure 5:**
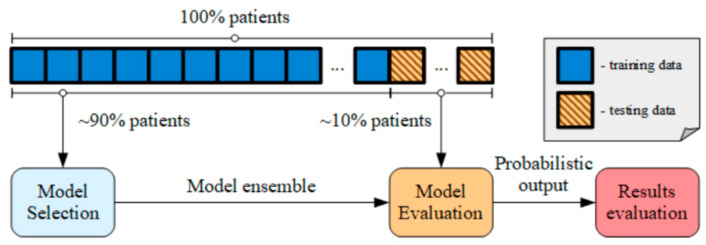
Model evaluation routine using 10-fold cross-validation.

Figure 5 shows the performance assessment and model selection methodology used in this work. To evaluate the model, a stratified 10-fold patient-independent cross-validation (CV) procedure was utilised. The whole dataset is split into 10 folds of similar sizes and a balanced representation of both classes, similar to the whole dataset (stratification). One of the folds is used for testing, whereas the others are used for training. The patient-independent model evaluation strategy aims to estimate the performance for an unseen patient [31] by distributing every data point belonging to a given patient into the same fold (train or test). 

### 2.6. Model Selection

**Figure 6:**
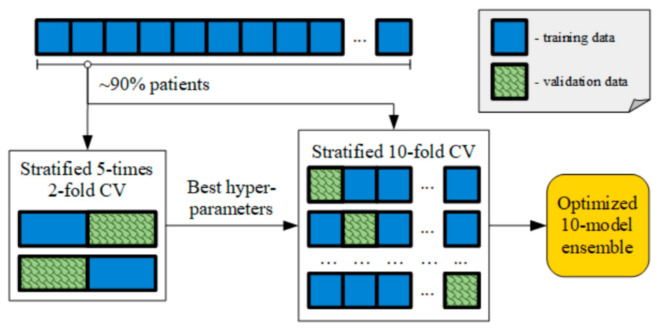
Model selection routine combining 5-times 2-fold cross-validation for hyperparameter selection and 10-fold cross-validation to obtain an optimised 10-model ensemble.

The usage of an ML algorithm requires a clearly defined and independent model selection routine [32]. The model selection aims to optimise a model over a set of hyperparameters to ensure the resultant model maintains its performance on unseen testing data. In this study, a nested CV procedure is utilised, as shown in Figure 6. 

The model is optimised with respect to the following hyperparameters: *max_depth*, *subsample*, *colsample_bytree*, *tree_num*. First, the three hyperparameters, *max_depth*, *subsample*, and *colsample_bytree*, are selected in a 5-times 2-fold CV (i.e., 2 folds are split 5 times with different random shuffling of the data) using out-of-fold data for assessment. After that, with those three hyperparameters now fixed to their optimal values, 10-fold CV is performed in order to generate an ensemble of 10 models optimised to the best number of boosting rounds (or the number of trees) by maximising the evaluation metric on the validation data set (early stopping).

### 2.7. Metric

The chosen metric to assess the performance of this study is the area under the curve (AUC) [33]. This metric is calculated from the array of predictions given by the model and the respective array of ground truth obtained from the ultrasound. The predictions for each cycle for each auscultation point are aggregated for the whole patient to lead one probability value per patient, which is then contrasted with the patient label.

### 2.8. Design of Experiments

For each patient, the physiological information comes for each cycle for each auscultation point. Each patient can be represented as a sequence of feature vectors. The ground truth is available for each patient but not for each feature vector. The information combinations at feature and decision levels are experimented to check the model accuracy for the chosen aggregation methodology.

At the feature level, the aim is to condense the information from all cycles into a single feature vector before the model. Two approaches are explored based on how the information given by the auscultation points is used. In the first approach, all cycle information is averaged separately per each auscultation point, condensing the patient’s information into just five feature vectors (one per each auscultation point). These five feature vectors are then concatenated into a single feature vector per patient. This first approach assumes that, if present, the audible signatures manifest in each cycle, but some auscultation points can be more important than others. In the second approach, the feature vectors are averaged across all the cycles and all the auscultation points. This approach considers that all patient’s cycles contain similar information, even across different auscultation points. 

When considering aggregation over multiple sources of information at the decision level (post-processing), the cycle-level predictions can be processed across each auscultation point first and then aggregated across patient using mean or maxima. All four possible cases are considered to determine if the decision needs to be done based on the common behaviour of features (mean) or on the oddities (max). The baseline performance is obtained by aggregating all predictions from all cycles and all auscultation points with mean with no feature transformation.

The experiments mentioned above were performed first for the task of PDA vs. healthy. This means that only the data from patients belonging to healthy and PDA groups was utilised to generate the binary classification mode, excluding CHD data instances. Once the best methodology is defined, it is also replicated for the task of CHD vs. healthy (i.e., utilising data from CHD and healthy patients, excluding PDA instances).

The feature importance is studied from the final model by quantifying each feature contribution to the classification task. The feature selection experiments are conducted to examine the performance with all features available vs. using just the Top-60, Top-30, Top-15, Top-10, and Top-5 features.

**Figure 7:**
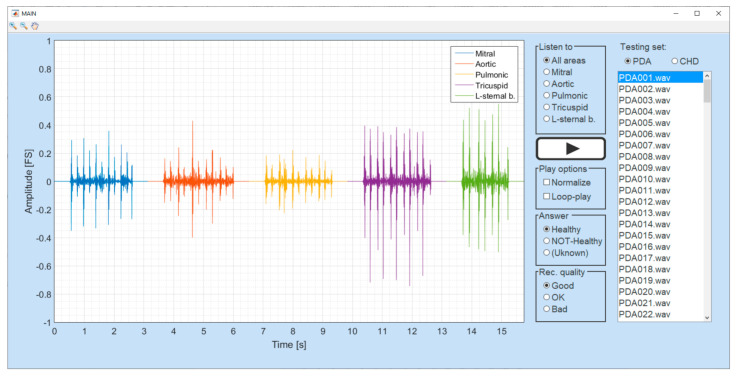
Graphic user interface for human performance assessment.

Finally, it is desired to know how well the ML model performs in comparison with a trained doctor performing the same task with access to audio-only. The human performance is tested as follows: the doctor was asked to determine whether a patient is healthy or has audible signatures of PDA while listening to the audio data acquired from the five auscultation points. To make this process user-friendly, a graphical user interface (GUI) was designed in Matlab, as shown in Figure 7. For each randomly chosen patient, the doctor was able to listen to all auscultation points consecutively or focus on just one of them. The doctor could also normalise the volume and play the recording in a loop. After listening, the doctor can tag the patient as healthy, not healthy (PDA), or not sure, with the latter indicating a lack of clear diagnosis. The quality of the recordings was also assessed subjectively by the healthcare professional according to the number of auscultation points in which external noises were present. Those external noises included mainly baby crying, people speaking, or movement artefacts (i.e., skin scratching). If just one out of the five auscultation points contained external noises, the recording was categorised as good quality; if those noises were present on four to five of the auscultation points, the recording was categorised as bad quality. Average quality was chosen when those noises were present in just two to three of the five auscultation points. The recording names were previously randomised and anonymised in order to prevent making decisions based on the order of the files or the file names. The answers are collected and processed to retrieve the experienced healthcare professional’s sensitivity and specificity to discriminate based on the sound only.

## 3. Results

Table 5 shows the developed XGBoost system performance for various combinations of model selection and model evaluation routines. The performance is presented for validation (data used for early stopping) and test data (completely unseen data). Setting 1 uses patient-independent splits both for internal and external CV loops, thus, keeping each patient’s integrity. This setting used as a baseline throughout the study shows validation and test AUCs of 0.761 and 0.743, respectively. Setting 2 shows the effect of a data leakage that occurs when the model-selection uses random split without keeping patient-integrity. Setting 3 shows the greater extent of the data leakage when the data are randomly split between train and test.

Table 6 and Table 7 show the performance for different ways of information aggregation on the feature and decision levels, respectively. The mean of feature vectors across patient achieves the best result.

Table 8 shows the performance of the two tasks considered. It can be seen that the detection of PDA is more challenging than the detection of CHD with the latter obtaining an AUC of 0.775.

Table 9 shows the performance while reducing the number of features. Top-N indicates that only the highest-ranked N features were used. Performance consistently increases as the number of features is reduced until Top-15 features where the performance stays within the CI95 limits.

**Figure 8:**
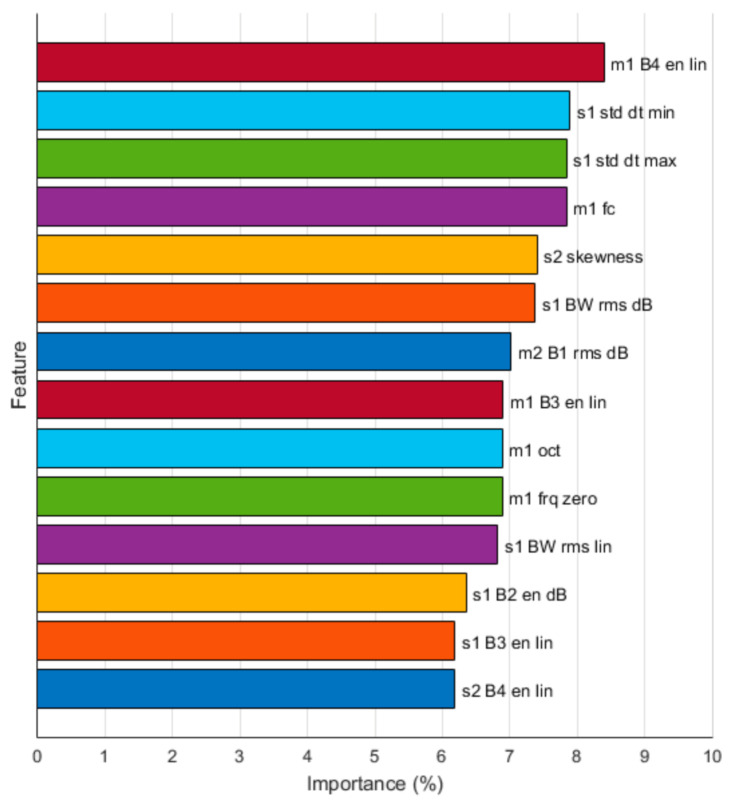
Top-15 features.

Figure 8 shows the Top-15 features. Features related to Systolic intervals (S1, m1) have a major impact on the classification task.

**Figure 9:**
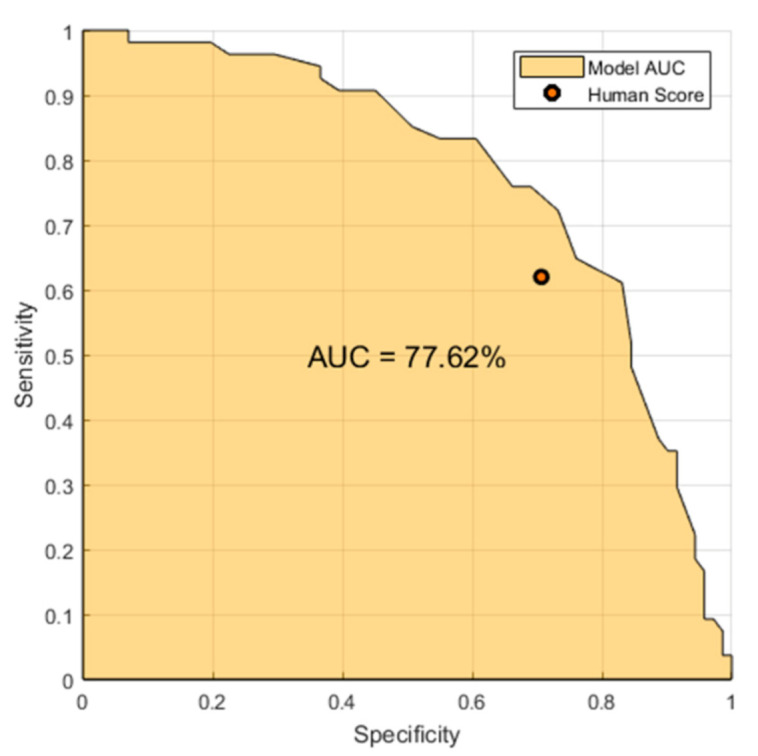
Best-model AUC vs. human sensitivity-specificity.

Figure 9 shows the model comparison (Top-15) and human obtained accuracy for the PDA vs. healthy task. The healthcare professional assessed randomly chosen 50% of patients. The AUC represents the performance of the model when evaluated on the same subset.

**Figure 10:**
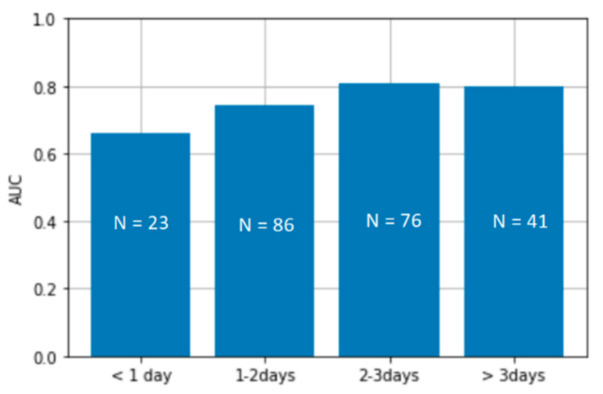
The PDA detection algorithm’s performance across cohorts of patients sorted by the days since birth when the recording was obtained.

To gain a more clinical insight into the model performance, Figure 10 shows the performance of the developed PDA detection algorithm when evaluated on the data sorted based on the days since birth when the recording was obtained. It can be seen that the worst performance is obtained on the recordings taken during the very first few days of life.

## 4. Discussion

A number of attempts have been made to differentiate normal and abnormal heart sounds in an adult [20,34] and paediatric populations [21] with various algorithms developed and features investigated. A few attempts to address a similar problem in a neonatal population [23,24] have concluded that the PCG assessment with ML was possible with several statistically significant features identified. However, these studies have been performed on a small cohort of newborns, and a human reference point was not provided. This study proposes an automated system for detection of CHD/PDA signatures in sound clips for the task of efficient prioritisation of candidates for ultrasound assessment to improve timely diagnosis and treatment in low-resource settings. To the best of our knowledge, it is the first study where the ability of the designed ML-based method to identify the presence of PDA signature in a sound clip is contrasted against both the ability of an experienced neonatologist to do the same as well as against the ultrasound gold standard labels.

### 4.1. Importance of Correct Model Selection and Evaluation Frameworks

When assessing the model’s performance, it is important to report the true generalisation error rather than the best achievable score. For the PDA/CHD detection systems to be useful in practice, their performance must hold on the unseen data. Moreover, the unseen data are expected to come from an unseen patient. Table 5 shows the true validation and test performance for Setting 1 when the performance was obtained in a patient-independent manner. The value of validation and test scores are very close, with an AUC of 0.761 and 0.743 for validation and test scores, respectively. 

When the validation loop is not conducted in a patient-independent manner (Setting 2), the models can be over-optimised, resulting in an overoptimistic assessment of the validation performance. The validation performance drives the selection of the model—which features to use, the hyperparameters of the model, pre-processing and post-processing routines. If it is not representative of the test performance, wrong choices can be made during the model development process. It can be seen from Table 5 that Setting 2 results in a bigger discrepancy between validation and test performance, in comparison to Setting 1. 

The accuracy of patient-dependent performance assessment (Setting 3) is much higher. This shows the unrealistically good score, which will not be achieved in practice on unseen patients. In practice, the algorithm is expected to perform on unseen patients [18,21,22,23,24,34]. However, these results can indicate the performance of the model for patients with follow-ups.

### 4.2. Combining Information Sources

Each patient’s physiological data in this study can be represented as a set of multiple information sources. Each recording consists of audio data from five auscultation points. Each auscultation point consists of multiple heart cycles, and each cycle can be further segmented to four different stages (s1, m2, s2, m2). A single recommendation has to be made for a patient who requires an algorithmic approach to aggregate over multiple information sources. Each feature was averaged across multiple cycles [23] to ensure that every cardiac cycle equally contributes to patient representation. Dynamic time warping has been used in [24], before feature extraction, to select the best-cycle to ensure that the chosen audio sample reflects the overall patient characteristics and does not contain outliers due to respiratory or movement artefacts, or other sources of occasional undesired noises. To the best of our knowledge, the combination of multiple auscultation points has been previously discussed neither for adults nor for paediatrics nor for neonatal cohorts. 

When comparing various ways to combine the available information sources at the level of features, shown in Table 6, it can be seen that a marginal improvement from an AUC of 0.761 to 0.763 (Validation scores) can be obtained with the method used in [23], namely, averaging each feature value across all available cycles. This indicates that each feature gets marginally more discriminative when averaged across each cycle even across different auscultation points (Table 6, Mean of features). Interestingly, when features are averaged within each auscultation point, and features from five points are concatenated (Table 6, Concatenation of features) the performance significantly degrades, dropping from an AUC of 0.761 to 0.666. This indicates that the location of audible signatures for each patient can be different, with some locations more important than others. However, these locations are patient-specific, and there are no learnable patterns that can generalise across all patients. 

When considering aggregation over multiple sources of information at the decision level through the post-processing, the methods based on the presence of oddities (taking max probability) either across cycles or across the auscultation points or both did not boost the performance. 

### 4.3. Features

This study intentionally utilises a large set of features from time and frequency domains to assess the level of their relevance for the considered tasks. Many of these features have been previously used for heart sound assessment [19,35]; others have been introduced here. It can be seen from Table 9, that both validation and test performance can be improved with the selection most relevant features. When comparing the performance obtained on the training data with the validation data, the large difference seen can be a sign of overfitting. This type of overfitting comes from the limited sample size and large feature set originally extracted. The reduced feature space improves the level of generalisation of the resultant models, which can be seen through the reduced discrepancy between training and validation performances.

The extracted features describe the data within four different segments of a cardiac cycle. It can be seen from Figure 8 that the majority of the most important features tend to describe selective frequency and energy content from m1 and s1 intervals. However, among the top 15 features, a few characteristics cover the other two segments of the cardiac cycle, m2 and s2. The best single most important feature appears to be the energy at the frequency range from 200 to 400 Hz from the systolic period (m1 B4 en lin). This result is in line with the findings in [23] where one of the most significant features for neonatal PDA detection was the relative maximal envelope value of the systolic period and the estimated length of the murmur.

### 4.4. Detection of PDA and Detection of CHD

Most of the reviewed literature evaluates the detectors of murmurs [21,24]. In contrast, while ultrasound can show the presence of PDA, audible signatures can be absent. The results from Table 8 indicates that the developed system can detect the presence of CHD to a better level of accuracy than the presence of PDA, with an AUC of 0.78 vs. 0.74, respectively. The results are obtained with the same feature set, which shows the validity of the chosen features for both tasks. 

It is interesting to observe the performance of PDA detection improves with the age of the patient. PDA can be intermittent during the first days of life [8] and the classification gets better on patients with 48 h after birth, as the PDA becomes transitionally permanent during this time. This should be taken into account by clinicians when choosing the time of examination of the newborn, especially if the discharge from the hospital coincides with this period.

### 4.5. Comparison with the Human Assessment

Comparing results obtained with different machine learning algorithms on different datasets in different setups is a challenging task. A point of reference for a given dataset and algorithmic solution can be established by comparing with the human accuracy obtained on the same dataset. In [14], the computer-assisted auscultation was contrasted with traditional auscultation for detection of murmurs on a cohort of 100 paediatric patients. Seven doctors listened to a set of recordings twice in randomised orders with the second time with computer provided probability of murmur presence. Traditional auscultation was shown to be outperformed by the computer-aided auscultation, improving both sensitivity and specificity, from 0.867/0.635 to 0.929/0.786, respectively. 

Figure 9 shows that the developed model marginally outperforms the human listener, improving the sensitivity from 0.62 to 0.72 for the same fixed specificity and improving the specificity from 0.71 to 0.82 for the same fixed sensitivity. The results indicate that the developed system has a strong potential to augment and support clinical decision making by providing a source of accurate and objective information.

Obtaining high-quality PCG from a newborn was difficult due to the child’s movements and discomfort. When possible, the recordings were made during the child’s sleep, sometimes through one layer of clothing. Thus, only 12% of the recordings examined by the health care professional were subjectively categorised as Good quality recordings. The rest were categorised as Bad (40%) or OK (48%). Approximately half of the data examined contained noises external to the heart sounds, making the task of discriminating PDA from healthy recordings more challenging for both ML and the healthcare professional on this particular dataset. Interestingly, no consistent dependency was observed between the quality of the recording and performance of either ML models or the human listener. 

### 4.6. Further Considerations

This study utilised a manually segmented dataset. An automatic segmentation algorithm that does not require human intervention is preferable to automatise the segmentation process. There are no existing segmentation algorithms developed for the neonatal population. The algorithms that are created for the adult population [36] could potentially be adapted to work on neonatal PCGs after accounting for faster heart rates in newborns. 

The features considered in this work are relatively simple and computationally inexpensive. The extraction of advanced features from frequency or information theory domains can also be explored. There have been few attempts to use deep learning to combine feature engineering and classification in one end-to-end optimisation for processing of adult PCG [34]. While superior in nature and well suited to audio signals, a deep learning approach will require a considerably larger amount of data to properly train the models. Additional data can result from automatic segmentation methods and from more data recorded during the clinical deployment of the algorithms. 

The screening task considered was to determine whether the patient is healthy or recommended for additional inspection (either PDA or CHD). Therefore, determining the exact diagnosis, the type of the CHD, or the quantity of the CHDs and their combinations was not addressed in this study. 

The procedure of dividing PDA, CHD, and healthy in two separate tasks, PDA/healthy and CHD/healthy, is a simplification as in real life PDA and CHD could co-occur in the same patient. 

While the study focuses on auscultation alone, a total clinical assessment is a multidimensional process. Clinical examination, including auscultation, and pulse oximetry (PO) are used for screening for CHD. PO estimates the blood oxygen saturation using differences in light absorption characteristics of oxygenated and deoxygenated haemoglobin [37]. If performed between 24–48 h after birth, the detection of those CHDs that affect the infant’s oxygen saturation, it is a screening method with moderate sensitivity and high specificity [38,39,40]. Whereas clinical examination alone is known to have limited sensitivity (77.4% (95% CI 70.0–83.4%)) the addition of PO leads to a significant increase in sensitivity (93.2% (95% CI 87.9–96.2%)) [41]. These results have been confirmed and found that the sensitivity of the combination of PO with auscultation is 95.5% (95% CI 84.9–98.7%) for critical CHD and 92.1% (95% CI 87.7–95.1%) for major CHD [42]. Through the assessment of the performance of the healthcare professional to detect the presence of PDA in the sound alone the study did not aim to underscore of the clinician’s ability to detect infants requiring echocardiography. Instead, the study aims to underline the added value of the objectivity that can be introduced with ML into the screening process, which might improve the screening accuracy overall. The usage of the developed tool in clinical bedside practise needs to be further evaluated in prospective trials. As PDA diagnosis mainly affects preterm infants, the performance of our algorithm should also be prospectively evaluated in a more immature cohort. 

In a subgroup of CHDs, the so-called ductus-dependent heart defects, the PDA needs to remain open. These defects might manifest clinically only during the functional closure of the ductus arteriosus, which may occur after the child is discharged home. The detected arterial duct before discharge can be a reason to refer the newborn for echocardiography [43]. The introduction of PO improved detection of PDA dependent CHDs to a total detection rate of duct dependent circulation to 92% [44]. The presented framework could only benefit from the addition of pulse oximetry data to further improve the decision-making process.

## 5. Conclusions

This work presents the development of an objective clinical decision support tool based on machine learning (ML) to facilitate differentiation of sounds with signatures of PDA/CHDs, in clinical settings. The solutions are validated on a comprehensive real clinical dataset composed of heart sound recordings from a total of 265 newborns. To the best of our knowledge, it is the first study where the ability of the designed ML-based method to identify the presence of PDA signature in a sound clip is contrasted against both the ability of an experienced neo-natologist to do the same as well as against the ultrasound gold standard labels. This study has the potential of an earlier and more reliable screening to efficiently use the available resources without putting infants at risk of missed diagnoses and delayed treatment. 

The future work will focus on assessing the difference between human performance and ML performance on the classification of various murmurs. The importance of pulse oximetry in improved detection of PDA and CHD suggests that integrating PO data in our framework will result in a more comprehensive assessment of the performance of artificial intelligence-augmented decision-making. The deployment of the models in real-life via cloud-based decision support will enable more data to analyse, more participation from medical professionals leading to a more accurate analysis. The developed models for PDA and CHD detection are in the process of being deployed in the cloud-based service www.hearttone.org.

## Figures and Tables

**Table 1:** Demographic breakdown of the dataset (N = 265).

Hospitals:	
− Centre 1	3 (1%)
− Centre 2	262 (99%)
Sex:	
− Males	137 (52%)
Gestational age [weeks]:	Median 39, IQR: 38–40
− Less than 37 weeks (preterm)	23 (9%)
− 37 weeks or more (term)	242 (91%)
Postnatal age [hours]	Median 48, IQR: 30–64
− Less than 24 h	29 (11%)
− From 24 to 48 h	95 (36%)
− From 48 to 72 h	87 (33%)
− More than 72 h	54 (20%)
Diagnostics	
− Healthy	137 (52%)
− Patent Ductus Arteriosus (PDA)	89 (33%)
− Other Congenital Heart Defects (CHD)	39 (15%)
− Ventriculoseptal defect	28 (71% of all CHD)
− Partial anomaly of pulmonary veins	6 (15% of all CHD)
− Congenital defect of trilateral valve development	2 (5% of all CHD)
− Atrioseptal defect	1 (3% of all CHD)
− Congenital aortic valve stenosis	1 (3% of all CHD)
− Tetralogy of Fallot	1 (3% of all CHD)
Total number of patients	265

**Table 2:** Features extracted from all four intervals of the heartbeat cycle.

Index	Tag	Description
1	energy	sum of squared values
2	n_zero	number of zero-crossings
For filtered signal 25–1000 Hz:		
3	bw_en_lin	energy as sum of squared values
4	bw_en_db	energy in dB scale
5	bw_rms_lin	root mean squared (RMS)
6	bw_rms_db	RMS in dB scale
For sub-bands (k) 25–45, 45–80, 80–200, 200–400, and 400–1000 Hz:		
7–10	bk_en_lin	energy as sum of squared values
11–14	bk_en_dB	energy in dB scale
15–18	bk_rms_lin	root mean squared (RMS)
19–22	bk_rms_dB	RMS in dB scale
23	fc	central frequency (<200 Hz)
24	oct	frequency deviation from average fc (per point), in octaves
25	trel	the relative length of the interval over the average length of the full cycle

**Table 3:** Features extracted only from S1 and S2 intervals of the heartbeat cycle (tagged as S1 and S2, respectively).

Index	Tag	Description
Absolute extrema:		
26	a_max	maximum value (positive amplitude)
27	t_max	relative time location of a_max
28	a_min	the minimum value (negative amplitude)
29	t_min	relative time location of a_min
30	max_a	maximum absolute value
31	max_t	relative time location of max_a
Local extrema:		
32	mean_t_max	mean time across all relative maxima
33	mean_dt_max	mean-time difference across all relative maxima
34	std_t_max	standard deviation over the time of all relative maxima
35	std_dt_max	standard deviation over the time difference between all relative maxima
36	n_max	number of local maxima
37	mean_t_min	mean-time across all relative minima
38	mean_dt_min	mean-time difference across all relative minima
39	std_t_min	standard deviation over the time of all relative minima
40	std_dt_min	standard deviation over the time difference between all relative minima
41	n_min	number of local minima
42	mean_t_zero	mean-time across all zero-crossing
43	std_t_zero	standard deviation over the time of all zero-crossing
44	mean_dt_zero	mean-time difference across all zero-crossing
45	std_dt_zero	standard deviation over the time difference between all zero-crossing
46	mean_t_max	mean-time across all relative maxima
Structural:		
47	n_broken	number of discontinuities on the derivative of the signal
48	skewness	the relative position of the maximum absolute value of the signal around the middle point of the interval

**Table 4:** Features extracted only from the systolic and diastolic silent intervals (tagged as m1 and m2, respectively).

Index	Tag	Description
Envelope approximation using 2nd order polynomial coefficients:		
49	a0	constant term of the polynomial
50	a1	linear term of the polynomial
51	a2	quadratic term of the polynomial
Energy distributed across time (4 quarters):		
52	en_1/4	1st quarter energy
53	en_2/4	2nd quarter energy
54	en_3/4	3rd quarter energy
55	en_4/4	4th quarter energy
Statistics:		
56	mean	mean value
57	std	standard deviation
Statistics distributed across time (4 quarters):		
58	mean_1/4	1st part mean
59	mean_2/4	2nd part mean
60	mean_3/4	3rd part mean
61	mean_4/4	4th part mean
62	std_1/4	1st part standard deviation
63	std_2/4	2nd part standard deviation
64	std_3/4	3rd part standard deviation
65	std_4/4	4th part standard deviation
Structural:		
66	frq_zero	number of zeros per second
67	skewness	relative position of the maximum absolute value of the signal around the middle point of the interval.

**Table 5:** Different settings over the baseline experiment for model evaluation and model selection.

	Model Evaluation	Model Selection	Validation (Area under the Curve (AUC)) (Mean ± Std)	Testing (AUC)
Setting 1	Pat. independent	Pat. independent	0.761 ± 0.004	0.743
Setting 2	Pat. independent	Pat. dependent	0.894 ± 0.000	0.749
Setting 3	Pat. dependent	Pat. dependent	0.876 ± 0.000	0.888

**Table 6:** The performance with the feature-level aggregation of information.

Feature Transformation	Dimensionality: (Datapoints, N Features)	Validation (AUC) (Mean ± Std)	Testing (AUC)
Baseline	(5904, 200)	0.761 ± 0.004	0.743
Concatenation of features	(265, 1000)	0.666 ± 0.004	0.648
Mean of features	(265, 200)	0.763 ± 0.003	0.754

**Table 7:** The performance with the decision-level aggregation of information.

Patient Combination	Point Combination	Validation (AUC) (Mean ± Std)	Testing (AUC)
Baseline		0.761 ± 0.003	0.743
Average	Maxima	0.750 ± 0.003	0.741
Maxima	Average	0.727 ± 0.003	0.706
Maxima	Maxima	0.715 ± 0.002	0.707

**Table 8:** The comparison of performance for PDA vs. healthy model and CHD vs. healthy model.

Detection Task	Validation (AUC) (Mean ± Std)	Testing (AUC)
PDA	0.761 ± 0.004	0.743
CHD	0.773 ± 0.002	0.775

**Table 9:** The performance with reduced feature sets for PDA vs. healthy.

Features Selected	Training (AUC) (Mean ± Std)	Validation (AUC) (Mean ± Std)	Testing (AUC)
Baseline (All features)	0.980 ± 0.003	0.761 ± 0.004	0.743
Top-60	0.961 ± 0.003	0.764 ± 0.004	0.754
Top-30	0.934 ± 0.006	0.768 ± 0.002	0.737
Top-15	0.877 ± 0.005	0.771 ± 0.003	0.776
Top-10	0.854 ± 0.004	0.774 ± 0.003	0.772
Top-5	0.847 ± 0.005	0.773 ± 0.002	0.777

## Data Availability

The data are not publicly available due to their sensitive and personal nature.
